# Salivary Function after Radioiodine Therapy: Poor Correlation between Symptoms and Salivary Scintigraphy

**DOI:** 10.3389/fendo.2015.00100

**Published:** 2015-06-17

**Authors:** Jacqueline Jonklaas, Hong Wang, Giuseppe Esposito

**Affiliations:** ^1^Division of Endocrinology, Georgetown University, Washington, DC, USA; ^2^Department of Biostatistics, Medstar Health Research Institute, Hyattsville, MD, USA; ^3^Department of Nuclear Medicine, Georgetown University, Washington, DC, USA

**Keywords:** salivary symptoms, salivary dysfunction, salivary scintigraphy, nasal symptoms, radioiodine therapy

## Abstract

**Objective:**

Symptoms of salivary gland dysfunction frequently develop after radioactive iodine (RAI) therapy, but have generally not been correlated with assessment of salivary gland functioning. The aim of this study was to determine whether there was a correlation between salivary symptoms and salivary functioning as assessed by salivary scan parameters.

**Methods:**

This was a non-randomized observational study. Fifteen patients receiving RAI therapy for differentiated thyroid cancer completed a questionnaire assessing their salivary and nasal symptoms prior to their therapy and 3 and 12 months after their therapy. Salivary gland scanning using technetium-99m pertechnetate was performed at the same time points. In addition, protective measures used at the time of radioiodine administration, such as use of fluids and sour candy, were also documented. Measures of salivary gland accumulation and secretion were correlated with scores of salivary and nasal symptomatology and any effects of protective measures were assessed.

**Results:**

The mean number of salivary, nasal, and total symptoms at 3 months increased significantly over the number of symptoms at baseline by 3.7, 2.7, and 6.3 symptoms, respectively (*p* values 0.001, 0.0046, and <0.001, respectively). The mean increases in the number of salivary, nasal, and total symptoms at 12 months were non-significant at 1.3, 1.3, and 2.5 symptoms, respectively. The mean right parotid gland accumulation and secretion of radioisotope declined significantly at 3 months, compared with baseline. The changes in left parotid and right and left submandibular function were non-significant. There was no association between the increase in salivary, nasal, or total symptoms and the change in scintigraphy measures. However, the increases in nasal and total symptoms were significantly greater in those with co-existent Hashimoto’s disease, compared with those without this condition (*p* values 0.01 and 0.04, respectively). Nasal symptoms decreased (*p* value 0.04) and total symptoms trended to decrease (*p* value 0.08) in those who used sour candies, compared with those who did not. Increasing body mass index was significantly associated with increasing nasal symptoms (*p* value 0.05). Greater decline in salivary parameters at 3 months compared with baseline was generally associated with heavier body weight, decreased thyroid cancer stage, absence of Hashimoto’s thyroiditis, and pre-menopausal status.

**Conclusion:**

Salivary and nasal symptoms increased and salivary scintigraphy parameters decreased after radioiodine therapy. However, the increased symptoms did not correlate with decrements in salivary gland accumulation or secretion. Moreover, the variables associated with symptoms and changes in salivary scan parameters differed. Therefore, a better understanding of the relationship between salivary gland symptoms and functioning is needed. Factors affecting susceptibility to salivary and nasal damage after radioiodine therapy need to be better elucidated, so that modifiable factors can be identified.

## Introduction

Administered radioactive iodine (RAI) is actively transported via the sodium iodide symporter and concentrated primarily by benign and malignant thyroid cells (1). Despite the proven efficacy of RAI in the treatment of thyroid cancer, there are several serious side effects associated with its use (2). These side effects, which are due to the impact of RAI on non-thyroid tissue, are one of the major causes of morbidity in thyroid cancer survivors (2, 3). Salivary glands are one of the most affected tissues.

The sodium iodide symporter has also been shown to be present in salivary tissue (1). Consequently, salivary glands have the ability to specifically concentrate iodine (1, 4), although its physiological role is not as yet elucidated. Salivary gland dysfunction after RAI treatment occurs quite frequently, even with a first therapy or low-dose therapy (5, 6). Salivary gland damage occurs in a dose-dependent manner but reported incidence rates vary widely from 5 to 86% (2, 7–10). More damage also seems to occur with treatment protocols involving withdrawal from thyroid hormone than with recombinant human thyrotropin (rhTSH) protocols (5). The parotid glands appear to suffer damage more frequently than the submandibular glands (4, 6, 10). Salivary gland dysfunction can present as sialadenitis, xerostomia, taste alterations, hypogeusia, sialolithiasis, dental caries, stomatitis, salivary gland or oral infections, facial nerve damage, and even salivary gland neoplasia (6, 9). Although side effects such as xerostomia may be transient, they may also persist for longer periods, be delayed in onset, or even be permanent (11, 12). Such events can profoundly decrease patient quality of life (2, 3).

Radioactivity has been demonstrated in tears (13) and contact lenses (14) after RAI administration, and the lacrimal system is another organ system affected by RAI. Lacrimal dysfunction can be manifest as epiphora, tearing, ocular dryness or xerophthalmia, and recurrent or chronic conjunctivitis. Xerophthalmia and abnormal lacrimal function tests were noted in 18 (9) and 92% (15) of patients, respectively after RAI therapy. Various rates of lacrimal dysfunction have been reported in other studies. These include conjunctivitis in 23% (16), significant tearing in 5% (17), “watery eyes” in 10% (18), and various symptoms of lacrimal dysfunction in 9.7% (19).

Nasal tissues are also affected by RAI. Nasopharyngeal tissues do not express the sodium iodide symporter (20), in contrast to the finding of its expression in extra-thyroidal tissues such the salivary glands (20) and lacrimal system (21). However, iodine may be transported non-specifically from the lacrimal system into the nasal ducts, and then from the interstitial surface of nasal ducts into the ductal lumen and out onto the nasal mucosal surface. A recent retrospective study (19) showed that the nasal side effects of RAI declared themselves within 1–2 weeks of the therapeutic dose. The mean time of onset of nasal symptoms was 11 days. The rate of nasal dysfunction was 10.5%: similar to the occurrence rate of 9.7% for lacrimal dysfunction. The nasal complaints included nasal pain, nasal tenderness, bloody nasal discharge, and dry nose. RAI dose and body mass index were significantly positively and negatively correlated with sustaining nasal side effects. Being prepared for treatment using a withdrawal protocol was associated with increased risk of nasal side effects.

This study was designed to prospectively study symptoms of salivary dysfunction after RAI therapy, along with simultaneously documenting objective measures of salivary gland functioning. Nasal symptoms were concurrently assessed.

## Materials and Methods

This was an observational, prospective, non-randomized study. The aim of this study was to determine whether there was a correlation between salivary symptoms and salivary functioning as assessed by salivary scan parameters. The study was approved by the Georgetown University Institutional Review Board.

Patients who were scheduled to undergo RAI treatment with I-131 for differentiated thyroid cancer were recruited for the study. Details of their thyroid cancer features, including thyroid cancer stage as defined using the National Thyroid Cancer Treatment Cooperative Study Group staging system (22, 23) were documented. Participation involved completion of two questionnaires about salivary and nasal symptoms respectively and undergoing a salivary gland scan using technetium-99m (^99m^Tc) pertechnetate performed prior to receiving RAI and 3 months and 12 months after RAI treatment. Participation thus involved completion of two questionnaires and a salivary scan on three separate occasions. The salivary gland questionnaire included 15 questions and is shown in Table 1. The nasal symptoms questionnaire included 16 questions and is shown in Table 2. Question number 12 on the nasal questionnaire asked about a dry mouth, as did question 1 on the salivary gland questionnaire. Question 12 from the nasal questionnaire was not included in the questionnaire score, but was used to help determine whether patients answered the questions consistently. Each positive response from the two questionnaires was assigned one point, thus the score for each questionnaire could potentially range from 0 to 15.

**Table 1:** **Salivary gland symptoms questionnaire**.

*PLEASE RESPOND TO THESE QUESTIONS BY CHECKING THEAPPROPRIATE BOX*		
**HAVE YOU EXPERIENCED THE FOLLOWING IN THE LAST 3 MONTHS?**	**YES**	**NO**
1. Dry mouth		
2. Altered taste		
3. Lack of taste		
4. Metallic or bitter taste		
5. Difficulty in swallowing dry foods (e.g., bread, crackers)		
6. Difficulty speaking		
7. Pain in the salivary glands in front of the ear		
8. Pain in the salivary glands under the jaw		
9. Painful mouth or ulcers in the mouth		
10. Pain with swallowing		
11. Swelling in the salivary glands in front of the ear		
12. Swelling in the salivary glands under the jaw		
**PLEASE ANSWER YES OR NO TO THE FOLLOWING QUESTIONS**	**YES**	**NO**
13. Have you had cavities filled by your dentist in the last 3 months?		
14. Have you needed to carry bottled water or drinks with you in the last 3 months?		
15. During the last 3 months have you routinely chewed gum or sucked candies?		

**Table 2:** **Nasal symptoms questionnaire**.

*PLEASE RESPOND TO THESE QUESTIONS BY CHECKING THEAPPROPRIATE BOX*		
**HAVE YOU EXPERIENCED THE FOLLOWING IN THE LAST 3 MONTHS?**	**YES**	**NO**
1. Feeling of generalized nasal discomfort		
2. Feeling of a dry nose		
3. Feeling that your nose is irritated		
4. Feeling of a hot or burning nose		
5. Feeling that the skin inside your nose is cracking		
6. Crusting in the nose		
7. Pain in the nose		
8. Noticed blood on tissue paper		
9. Experienced nose bleeds		
10. Noticed an altered sense of smell		
11. Been bothered by dry eyes		
12. Been bothered by a dry mouth		

**PLEASE ANSWER YES OR NO TO THE FOLLOWING QUESTIONS**	**YES**	**NO**
13. Have you used saline nasal sprays in the last 3 months?		
14. Have you used any kind of ointments in your nose?		
15. Have you needed to use a vaporizer or humidifier in your home in the last 3 months?		
16. Have you used any kind of nasal rinses during the last 3 months?		

Salivary gland function was assessed using salivary gland scintigraphy. Scintigraphy was performed following intravenous injection of 8 mCi (296 MBq) of ^99m^Tc-pertechnetate. Following isotope administration, the patient was placed supine with the head slightly extended. A support was placed behind the patient’s neck and to the sides of the head to minimize head motion. Camera settings included a 128 × 128 matrix with a 2.67 zoom, energy window at the 140 KeV ^99m^Tc photopeak with 15% symmetric window. A high-resolution parallel hole collimator was used. Sequential parotid and sublingual gland images were acquired every 20 s for 20 min. Two milliliters of a 100% lemon juice solution were then administered with a syringe and images were acquired at the same frame rate for a further 20 min. Regions of interest were drawn for the parotid and submandibular glands. Regions of interests for the mouth and the respective backgrounds (temporal area for parotid gland and submental area for submandibular gland) were also obtained. Background-corrected time activity curves were then derived. Parotid and sublingual gland functioning were assessed in the same study. The general pattern of radiotracer changes during salivary scanning was as follows. Immediately after radiotracer administration, tracer accumulation in the major salivary glands was observed, with more accumulation in the parotid than in the submandibular glands. After lemon juice administration, rapid secretion of activity from all salivary glands was observed, with nadir levels reached within 3–4 min from the start of stimulation. This washout of activity was greater in the parotid than in the submandibular glands.

Two parameters were calculated: accumulation (uptake) of ^99m^Tc-petechnatate and secretion (excretion) of ^99m^Tc-petechnatate. Accumulation was defined as the ratio of gland to background counts at the point of maximum counts. Secretion was defined as the ratio of gland to background counts at the nadir achieved after lemon juice stimulation subtracted from the ratio of gland to background counts at the point of maximum counts. The methodology used was similar to that described by Aung and colleagues (24). Accumulation and secretion of both the right and left salivary glands were obtained prior to RAI treatment as a baseline for each individual patient. The baseline values were compared with those obtained at the two time points after RAI treatment. These time points after RAI therapy were 3 months and 12 months. The 3-month background-corrected uptake ratio and the 12-month background-corrected uptake ratio were each subtracted from the background-corrected uptake ratio at baseline in order to generate the change that occurred following RAI therapy.

During the study period, the isotope used for diagnostic RAI scanning was I-123, with I-131 used for therapy. None of the conditions surrounding the patient’s RAI therapy were manipulated because of study participation. Patients received the RAI activity and used the method of TSH elevation prescribed by their treating physician. In addition, recommendations in place regarding the use of salivary stimulants and hydration were those given by the prescribing physician and/or nuclear medicine physician. During the period of the study, patients were routinely admitted to an isolation room in the hospital for 22 h after receiving their RAI therapy. Patients were permitted to drink fluids, use sialogogues, and eat 2 h after RAI administration. As part of the protocol, use of lemon candy, use of fluids, and urination frequency were carefully documented. With respect to lemon candy, the type of lemon candy, amount, frequency, and duration of its use was documented. Similarly for fluid intake, the type of fluid consumed, and the amount, frequency, and duration of its use, was documented. Patients were also asked to quantify the length of time for which they consumed more than their usual volume of fluids. For urine output, the frequency of voiding during the initial 22 h after RAI treatment was recorded.

The baseline characteristics of study population were calculated. Due to the very small sample size (*n* = 15), the assumption of normal distribution could not be assessed. Non-parametric tests without assumption of distribution were used for the analysis. The Spearman correlation coefficient was used to assess the correlation among symptom score, scan results, and continuous patient variables. The Wilcoxon rank sum test or Kruskal–Wallis test was used to compare the distribution of symptom score and scan results between the categories of patient variables.

## Results

Patients were treated with RAI and underwent salivary scanning during the period February 2009 to June 2010. Fifteen participants were consented for the study. All 15 patients completed the baseline and 3-month symptom questionnaire and salivary scan. Funding was only sufficient for eight patients to complete the 12-month scan. The same eight patients also completed the symptom questionnaires at 12 months. The question used to assess the reliability of response to the questions was consistently answered in all cases. The characteristics of the participants are displayed in Table 3. The mean age of the patients was 45.7 years and 73% of them were female. The relevant details about the patient thyroid cancer characteristics are shown in Table 4A. The majority of patients had papillary thyroid cancer of typical histology. Approximately, 50% had multifocal disease and/or cervical lymph node involvement. One patient had distant metastases.

**Table 3:** **Characteristics of study participants (number = 15)**.

Characteristic (continuous variables)		Mean (SD)	Median (Q1–Q3)
Age (years)		45.7 (14.9)	42.0 (33.0–55.0)
Weight		81.5 (15.8)	76.0 (69.0–86.0)
BMI		28.3 (5.4)	26.8 (24.2–32.9)

**Characteristic (categorical variables)**		**Number**	**Percentage**

Sex	*Female*	11	73
	*Male*	4	27
Menopausal status	*Pre-menopausal*	6	40
	*Post-menopausal*	5	33
	*n/a*	4	27
Co-existent Hashimoto’s	*No*	11	73
	*Yes*	4	27

**Table 4:** **Parameters related to differentiated thyroid cancer and radioiodine treatment**.

**A. THYROID CANCER-RELATED PARAMETERS**			

Characteristic		Number	Percentage
Type of DTC	*PTC*	11	73.3
	*fvPTC*	3	20
	*FTC*	1	6.7
Multifocal	*Yes*	9	60
	*No*	6	40
Cervical node involvement	*Yes*	7	46.7
	*No*	8	53.3
Vascular or capsular invasion	*Yes*	4	26.7
	*No*	11	73.3
Extra-thyroidal extension	*Yes*	3	20
	*No*	12	80
Distant metastases	*Yes*	1	6.7
	*No*	14	93.3
DTC stage	*I*	6	40
	*II*	6	40
	*III*	3	20

**B. RADIOIODINE-RELATED PARAMETERS**			

**Characteristic (continuous variables)**		**Mean (SD)**	**Median (range)**

Radioiodine activity (mCi of I-131) [mBq]		126 (29) [4.6 (1.1)]	106 (100–182) [3.9 (3.7–6.7)]
TSH value (mIU/L)		113 (41)	98 (40–205)
Length of time that lemon candies were used (*in subset using candies*) (hours)		27 (24)	22 (8–94)
Number of lemon candies per hour during first 22 h (*in subset using candies*)		2.3 (1.1)	2 (1–4)
Length of time participants tried to increase hydration (hours)		36.9 (27.6)	22 (12–120)
Volume of fluid per hour during first 22 h (fluid ounces) [mL]		10.6 (3.2) [313.5 (94.6)]	10 (4–16) [295.7 (118.3–473.2)]
Number of urinations while awake during first 22 h		7.5 (2.3)	8 (3–12)
Number of times awoke from sleep to urinate during first 22 h		3.3 (1.8)	3 (1–8)

**Characteristic (categorical variables)**		**Number**	**Percentage**

Method of TSH elevation
*rTSH*		12	80
*Withdrawal*		3	20
Use of lemon candy
*Yes*		11	73
*No*		4	27

Twelve out of 15 patients were prepared for RAI administration using a recombinant rhTSH protocol. Details regarding RAI administration and attendant use of sialogogues and fluids are shown in Table 4B. Mean and median TSH values for all 15 patients were 113 and 98 mIU/L, respectively. Mean and median RAI activities administered were 126 mCi (4.6 GBq) and 106 mCi (3.9 GBq), respectively. Patients attempted to drink more than their usual volume of fluids for a mean of 36.9 h. The mean volume of fluid consumed during the initial 22 h of participant hospitalization was 10.6 fluid ounces (313.6 mL) per hour. Fluids consumed included water, orange juice, apple juice, lemon juice, and Gatorade^®^. Eleven out of 15 patients used sour candies following RAI administration. Those that used lemon candies used a mean of 2.3 candies per hour during the initial 22 h. The mean length of lemon candy use was 27 h. The lemon candies used included the following: Lemonheads, Brachs, Jolly Ranchers, Sour Beans, Citron, sour gummies (various brands), Sour Patch, and Warheads. During their inpatient stay, participants urinated a mean of 7.5 times during waking hours and 3.3 times during the night.

**Figure 1:**
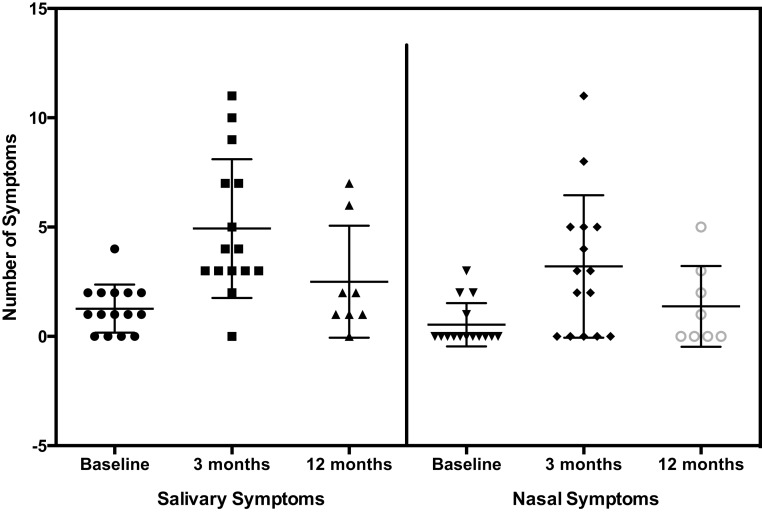
**Symptoms**.

The number of salivary and nasal symptoms reported by patients at baseline, 3 months, and 12 months are shown in Figure 1. The mean increases in the number of symptoms at 3 and 12 months over baseline are shown in Table 5A. The mean number of salivary, nasal, and total symptoms at 3 months increased significantly over the number of symptoms at baseline by 3.7, 2.7, and 6.3 symptoms, respectively (*p* values 0.001, 0.0046, <0.001). The mean increases in the number of salivary, nasal, and total symptoms at 12 months were non-significant at 1.3, 1.3, and 2.5 symptoms, respectively.

**Table 5:** **Symptoms developing after radioiodine treatment and associated variables**.

**A. INCREASE IN SYMPTOMS OVER BASELINE**				

Type of symptoms	Mean (SD)	*p* Value	Median (Q1–Q3)	Range
**Salivary**
Increase over baseline at 3 months	3.7 (2.7)	0.001	3.0 (1.0–7.0)	0–8
Increase over baseline at 12 months	1.3 (2.1)	NS (0.12)	0.0 (0.0–2.5)	0–5
**Nasal**
Increase over baseline at 3 months	2.7 (3.1)	0.0046	2.0 (0.0–3.0)	0–11
Increase over baseline at 12 months	1.3 (1.6)	NS (0.06)	0.5 (0.0–2.5)	0–4
**Total**
Increase over baseline at 3 months	6.3 (4.3)	<0.001	6.0 (3.0–10.0)	0–14
Increase over baseline at 12 months	2.5 (3.5)	NS (0.06)	1.0 (0.0–5.0)	0–8

**B. VARIABLES ASSOCIATED WITH INCREASE IN SYMPTOMS OVER BASELINE**				

**Type of symptoms**	**Categorical variables (yes vs. no)**	**Symptoms in patients with variable vs. without Mean (SD)**		***p* Value**

Increase in nasal symptoms at 3 months	Co-existent Hashimoto’s	6 ± 3.8 vs. 1.5 ± 1.6		0.01
	Use of lemon candy	1.5 ± 1.7 vs. 5.8 ± 4.1		0.04
Increase in total symptoms at 3 months	Co-existent Hashimoto’s	10 ± 3.4 vs. 5 ± 3.9		0.04
	Use of lemon candy	5.1 ± 3.9 vs. 9.8 ± 3.8		0.08

**Type of symptoms**	**Continuous variables**	**Spearman correlation**		***p* Value**

Increase in nasal symptoms at 3 months	BMI	0.665		0.05
Increase in nasal symptoms at 3 months	Duration of increased hydration	0.767		0.01

**Figure 2:**
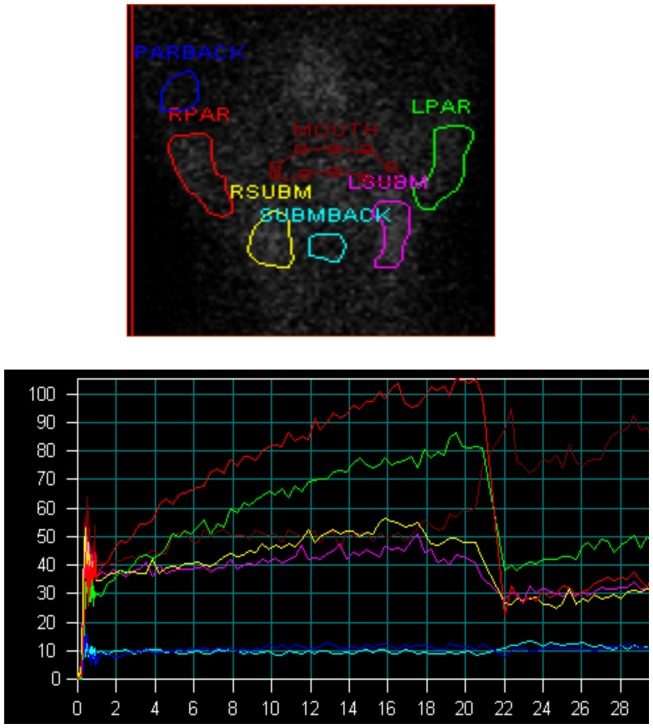
**Salivary scintigraphy**. Upper figure shows regions of interest, lower figure shows time activity curves (*y* axis = counts per second, *x* axis = minutes, lemon juice was administered at the 20 min time point). Red = right parotid, green = left parotid, yellow = right submandibular gland, pink = left submandibular gland, blue = parotid background, turquoise = submandibular background, brown = mouth.

**Figure 3:**
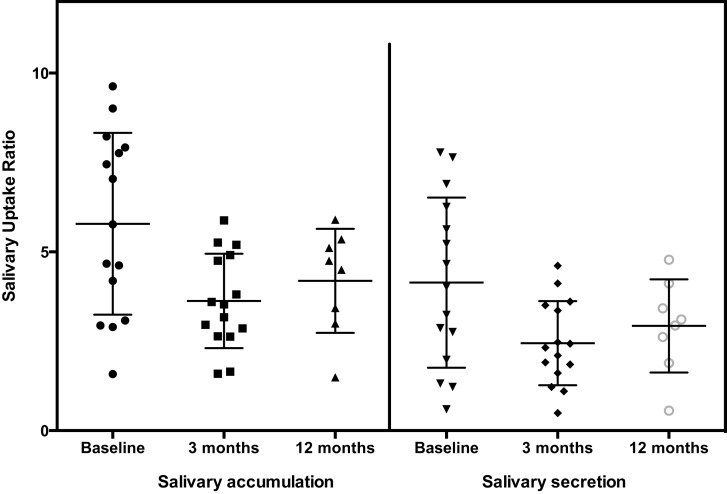
**Right parotid**.

**Figure 4:**
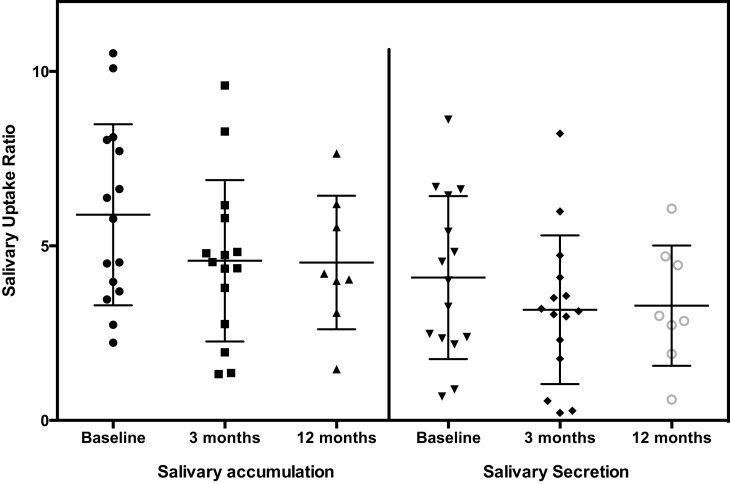
**Left parotid**.

The general pattern of radiotracer changes during salivary scanning was described in the Section “Materials and Methods.” A representative example of salivary gland accumulation and secretion is shown in Figure 2. The mean parotid gland accumulation and secretion of radioisotope generally declined at 3 months, compared with baseline. The declines in right parotid accumulation and secretion at 3 months were significant (see Table 6A; Figure 3). The changes in left parotid accumulation and secretion at 3 months, however, were not significant (see Table 6A; Figure 4). None of the changes in submandibular functioning or parotid gland functioning at 12 months, were significant (see Table 6A.) All the changes in salivary function were characterized by great variability, as can be seen in Table 6A and Figures 3 and 4.

**Table 6:** **Changes in salivary scintigraphy after radioiodine treatment**.

**A. CHANGE IN SALIVARY PARAMETER**						

Salivary gland parameter (expressed as uptake ratio)	Change at 3 months compared with 0 months			Change at 12 months compared with 0 months		
	**Mean**	**SD**	***p* Value**	**Mean**	**SD**	***p* Value**
**Salivary accumulation**
R. parotid	2.16	2.58	0.006	1.69	2.50	0.09
R. submandibular	−0.76	2.03	NS	−0.91	2.67	NS
L. parotid	1.32	3.31	0.15	1.03	2.19	0.23
L. submandibular	−0.27	2.43	NS	−0.57	1.55	NS
**Salivary secretion**
R. parotid	1.69	2.54	0.022	1.03	2.23	0.23
R. submandibular	−0.78	2.03	NS	−0.96	2.61	NS
L. parotid	0.92	3.23	0.28	0.29	1.85	0.67
L. submandibular	−0.28	2.34	NS	−0.58	1.50	NS

**B. VARIABLES ASSOCIATED WITH CHANGE IN SALIVARY PARAMETERS AT 3 MONTHS COMPARED WITH 0 MONTH**						

**Salivary gland parameter (expressed as uptake ratio)**	**Categorical variables**			**Change in parameter with vs. without**		***p* Value**

R. parotid accumulation	Weight ≤76 vs. >76 kg			0.9 ± 28 vs. 3.5 ± 1.5		0.05
	Stage TCR I vs. II vs. III			3.4 ± 2.1 vs. 2.3 ± 2.5 vs. −0.6 ± 2.1		0.06
R. parotid secretion	Weight ≤76 vs. >76 kg			0.5 ± 2.8 vs. 3.0 ± 1.5		0.07
	Stage TCR I vs. II vs. III			3.1 ± 2.3 vs. 1.6 ± 2.1 vs. −1.0 ± 1.8		0.06
L. parotid accumulation	F preM vs. postM			2.9 ± 3.1 vs. −1.2 ± 3.0		0.03
	Co-existent Hashimoto’s (yes vs. no)			−1.6 ± 3.4 vs. 2.4 ± 2.7		0.05
	Stage TCR I vs. II vs. III			3.1 ± 2.8 vs. 1.1 ± 3.5 vs. −1.8 ± 1.2		0.05
L. parotid secretion	F preM vs. postM			2.6 ± 3.0 vs. −1.4 ± 2.9		0.03
	Co-existent Hashimoto’s (yes vs. no)			−1.7 ± 3.3 vs. 1.9 ± 2.8		0.05
	Stage TCR I vs. II vs. III			2.7 ± 2.9 vs. 0.4 ± 3.4 vs. −1.7 ± 1.4		0.05

**Salivary gland parameter (expressed as uptake ratio)**	**Continuous variables**			**Spearman correlation**		***p* Value**

R. parotid accumulation	No of lemon candies/hour			−0.69		0.03
R. parotid secretion	Times urination day			−0.63		0.06
L. parotid accumulation	Volume/hour			−0.61		0.08
	Times urination day			−0.58		0.09
L. parotid secretion	Volume/hour			−0.64		0.06

There was no association between the increase in salivary, nasal, or total symptoms and the change in scintigraphy measures. However, the increases in nasal and total symptoms were significantly greater in those with co-existent Hashimoto’s disease, compared with those without this condition (*p* values 0.01 and 0.04, respectively) (see Table 5B). The increase in nasal symptoms was of lesser magnitude (*p* value 0.04), and the increase in total symptoms trended to be less (*p* value 0.08), in those who used sour candies, compared with those who did not. Body mass index was significantly associated with increasing nasal symptoms at 3 months (*p* values 0.05). Increased nasal symptoms at 3 months were positively associated with duration of increased hydration (*p* value 0.01) (see Table 5B). With respect to the categorical variables that were associated with greater decrement in salivary parameters, these included increased body weight (*p* value 0.05), lower thyroid cancer stage (*p* value 0.05), pre-menopausal status (*p* value 0.03), and absence of Hashimoto’s disease (*p* value 0.05) (see Table 6B). The only continuous variable with a significant association was fewer lemon candies per hour being associated a greater decrement in parotid gland accumulation at 3 months (*p* value 0.03). In this small study, neither symptoms nor scintigraphy measures appeared to be affected by the method of TSH elevation or the RAI dose administered.

## Discussion

This study showed an increase in symptoms of salivary gland dysfunction 3 months following RAI treatment followed by a decrease in symptoms by 12 months. At the same time, there was a decline in scintigraphy parameters that reflect parotid salivary gland functioning. Both accumulation and secretion of radiotracer were affected, with the right parotid being more affected than the left parotid. However, surprisingly, there was lack of correlation between salivary symptoms and scintigraphy measures of salivary function.

This is in contrast to a recent study in which all eight patients who had oral symptoms consistent with salivary dysfunction 12 months after RAI therapy were reported to have their salivary dysfunction confirmed by salivary scintigraphy (25). However, the particular deficit noted on scintigraphy (parotid vs. submandibular gland, accumulation vs. secretion) was not reported. However, another study also did not show correlation between symptoms and objective measures of salivary function (26). In this study of patients who received or did not receive RAI therapy, salivary scintigraphy parameters or salivary flow measured by sialometry were not associated with xerostomia or dysphagia (26). However, dysphagia and reduced parotid secretion were each associated with RAI therapy. An additional study examining correlation of symptoms with objective measures of salivary function in patients with thyroid cancer found that xerostomia was correlated with the number of salivary glands showing worsening scintigraphy parameters and that xerostomia correlated better with scintigraphic measures of submandibular dysfunction than parotid dysfunction (27).

The failure of symptoms of salivary function to correlate with salivary parameters in this study is most likely due to under-powering of the study. However, given that similar lack of correlation has been reported in other studies too (26, 27), perhaps additional factors contributing to symptomatology need to be investigated. The lack of correlation between symptoms and function may also be due to failure to collect data about other more pertinent symptoms that perhaps better mirror functioning. If true, this highlights the need for better, validated symptom questionnaires.

Variables associated with either salivary symptoms or scintigraphy changes have been elucidated. Higher activities of RAI have been associated with a greater frequency of xerostomia and greater changes in scintigraphy parameters (27). Another study identified patient age as being associated with decreased parotid accumulation and decreased salivary flow, whereas having received RAI therapy was associated with decreased parotid secretion and decreased salivary flow (27). In this study, the variables that were associated with increased salivary symptoms were different, and even of opposite impact, than the variables associated with a decline in salivary scan parameters. By way of illustration, co-existent Hashimoto’s thyroiditis was associated with an increase in symptoms at 3 months but a lesser decline in parotid parameters at 3 months. It is possible that the lesser decline in salivary function in patients with Hashimoto’s occurred because some dysfunction already existed prior to RAI due to underlying autoimmunity. In this present study, use of lemon candy, which has been shown in another study by Kulkarni and coworkers to decrease the radiation dose absorbed by the salivary glands (28), was associated with less nasal symptoms and less total symptoms. A greater number of lemon candies used per hour was also associated with less decrement in right parotid accumulation at 3 months in the present study. BMI and weight were positively associated with number of symptoms and greater decrement in salivary parameters; advancing thyroid cancer stage was associated with lesser decline in salivary parameters. If confirmed, these findings could be associated with altered handling of iodine associated with body composition, and the proportion of RAI taken up by non-thyroid tissue vs. residual thyroid cancer.

In confirmation of other studies, we show that there is a lesser effect of RAI therapy on submandibular functioning (4, 6, 10). The greater impact of ionizing radiation on the parotid glands is thought to due to their greater activity and greater content of serous cells, compared with the mixed serous and mucinous cell content of the submandibular glands (6). We are not aware of prior studies that suggest a greater effect of RAI therapy on the right parotid, as opposed to the left. Most studies report combined bilateral salivary gland parameters, but one prior study that reported separate function for right and left glands did not report any differential effect (27).

This study also gives new information about the duration of nasal symptoms after radioiodine therapy. A prior retrospective study has shown a mean time of onset of nasal symptoms of 11 days and no documentation of continuing symptoms from chart review of 3-month and 12-month endocrinology follow-up visits (19). This study, however, shows that, as demonstrated by questionnaire completion during a prospective study, nasal symptoms were still clearly present at 3 months.

The limitations of this study include the very small sample size, a non-validated questionnaire, lack of a control group not treated with RAI, and lack of randomization. To illustrate the need for a control group who did not receive RAI, some salivary scintigraphic parameters were more robust at 3 months than at baseline; a control group would have given insight as to whether this reflected a natural variation in salivary function and/or in the measurement techniques used. However, a positive aspect of the study is that the data were collected prospectively.

There is a clear need for better understanding of the cause of symptoms of salivary dysfunction after RAI administration. If these symptoms are confirmed in additional larger studies not to correlate well with objective measures of salivary functioning, then an investigation of potential alternative causes of these symptoms is needed. In addition, risk factors for both symptoms and altered scintigraphy parameters need to be better documented, especially given the opposing effects of some of these risk factors documented here.

## Conflict of Interest Statement

The authors declare that the research was conducted in the absence of any commercial or financial relationships that could be construed as a potential conflict of interest.
